# Cardiorenal ketone metabolism in healthy humans assessed by ^11^C-acetoacetate PET: effect of D-β-hydroxybutyrate, a meal, and age

**DOI:** 10.3389/fphys.2024.1443781

**Published:** 2024-10-21

**Authors:** Valérie St-Pierre, Gabriel Richard, Etienne Croteau, Mélanie Fortier, Camille Vandenberghe, André C. Carpentier, Bernard Cuenoud, Stephen C. Cunnane

**Affiliations:** ^1^ Research Centre on Aging, Sherbrooke, QC, Canada; ^2^ Sherbrooke Molecular Imaging Center, Sherbrooke, QC, Canada; ^3^ Centre de recherche du CHUS, Sherbrooke, QC, Canada; ^4^ Department of Medicine, Université de Sherbrooke, Sherbrooke, QC, Canada; ^5^ Nestlé Health Science, Vers chez les blancs, Vevey, Switzerland

**Keywords:** heart, kidney, metabolism, ketones, cardiorenal, positron emission tomography, acetoacetate, β-hydroxybutyrate

## Abstract

The heart and kidney have a high energy requirement, but relatively little is known about their utilization of ketones as a potential energy source. We assessed the metabolism of the ketone tracer, carbon-11 acetoacetate (^11^C-AcAc), by the left and right ventricles of the heart and by the kidney using positron emission tomography (PET) in n = 10 healthy adults under four experimental conditions: a 4-h fast (fasted) ± a single 12 g oral dose of D-beta-hydroxybutyrate (D-BHB), and a single complete, liquid replacement meal (hereafter referred to as the “fed” condition) ± a single 12 g oral dose of D-BHB. Under these experimental conditions, the kinetics of ^11^C-AcAc metabolism fitted a two-compartment model in the heart and a three-compartment model in the kidney. Plasma ketones were about 10-fold higher with the oral dose of D-BHB. During the four conditions, tracer kinetics were broadly similar in the myocardium and kidney cortex. ^11^C-AcAc metabolism by the kidney pelvis was similar in three of the four study conditions but, later, peaked significantly higher than that in the cortex; the exception was that the tracer uptake was significantly lower in the fed condition without D-BHB. ^11^C-AcAc uptake was significantly inversely correlated with age in the kidney cortex, and its oxidative metabolism was significantly positively correlated with age in the left ventricle. D-BHB blunted the insulin, gastric inhibitory peptide, and C-peptide response to the meal. This PET methodology and these acute metabolic perturbations would be suitable for future studies assessing cardiorenal ketone metabolism in conditions in which heart and kidney functions are experimentally modified or compromised by disease.

## Graphical Abstract

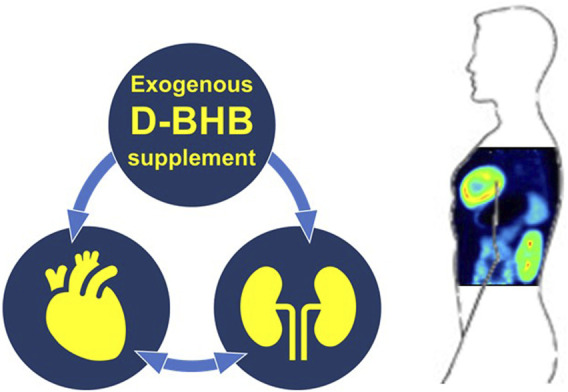

## Introduction

**FIGURE 1:**
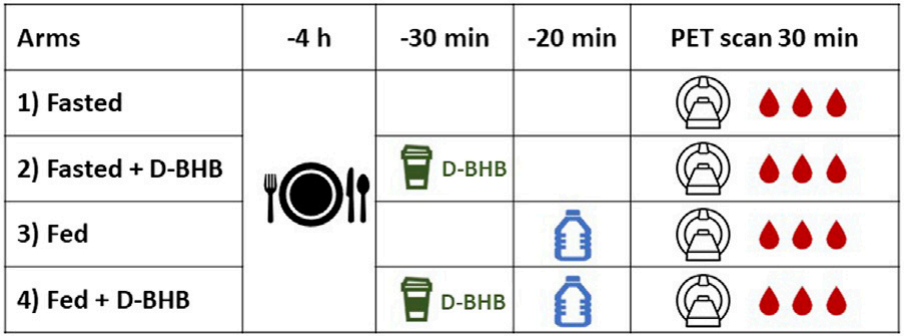
Overview of the study design. Each of the four conditions (1–4) was separated by at least 1 week. D-BHB: a single 12-g dose of D-β-hydroxybutyrate; fasted: nothing to eat for 4 h; fed: complete liquid meal (Boost^®^). Blood samples were taken during the dynamic positron emission tomography (PET) scan.

We recently reported the metabolism of the ketone body, acetoacetate (AcAc), by the heart and kidney in healthy humans using positron emission tomography (PET) (Cuenoud et al., 2023). The PET tracer for AcAc, ^11^C-AcAc, was metabolized by the myocardium in a manner quite similar to that of ^11^C-acetate (^11^C-Ac). However, in the kidney cortex, the fractional uptake (*K*_*1*_) was twice as high for ^11^C-Ac as for ^11^C-AcAc, while oxidative metabolism (*k*_*2*_) for ^11^C-Ac was 30% lower than for ^11^C-AcAc (Cuenoud et al., 2023). We now assessed, for the first time, the effect of three acute physiological variables (Figure 1)—short-term fasting (fast), a single liquid meal (fed), and with or without the consumption of a single oral dose of a D-β-hydroxybutyrate salt (D-BHB)—on the metabolism of ^11^C-AcAc by the heart and kidney in healthy humans over an age range of 35–70 years. We also assessed the effect of age on ^11^C-AcAc cardiorenal metabolism. The fed condition transiently increased the heart rate and cardiac output (Sundell and Knuuti, 2003; Scognamiglio et al., 2005), so it was of interest to assess the impact of the fed condition with or without an oral dose of D-BHB on these parameters. These results may provide an insight into whether an exogenous ketone such as D-BHB could be therapeutically beneficial for individuals in whom cardiac or renal function diminishes with age or in chronic conditions that change organ energy metabolism, such as heart failure and chronic kidney disease. These acute physiological experiments also served to better validate the kinetic model for ^11^C-AcAc metabolism since little is known about cardiorenal ketone metabolism.

The metabolism of both ^11^C-AcAc and ^11^C-Ac by the heart appears to broadly follow a two-compartment model, whereas significant AcAc excretion by the kidney to urine required a three-compartment model. Given the importance of these experiments to validate the kinetic model without having studied long-term interventions or disease conditions, we intentionally kept the interpretation to a minimum. Nevertheless, these physiological conditions provide a basis for further studies to understand more about the mechanisms involved.

## Materials and methods

### Study approval

The study protocol was approved by the Human Ethics Committee of the CIUSSS de l’Estrie-CHUS. All participants provided written informed consent in accordance with the Declaration of Helsinki. This study was registered at ClinicalTrials.gov (NCT04580823) under the name “Heart and Kidney Ketone Metabolism.”

### Participant selection

Ten participants were recruited for this study by advertising. Inclusion criteria were (i) age 35–70 years old; (ii) BMI between 18.5 and 30 kg/m^2^; and (iii) a good general health or chronic health condition with stable medication for ≥3 months. The main exclusion criteria were (i) medications influencing blood glucose and insulin levels, thyroid function, and renal or cardiac metabolism; (ii) smoking; (iii) pregnancy or lactation; (iv) diabetes or pre-diabetes (fasting glucose >6.1 mM and glycosylated hemoglobin >6.0%); (v) intense structured physical activity (>3 times/week); and/or (vi) consumption of a ketogenic diet.

### Experimental design and patient preparation

The participants were instructed to abstain from alcohol and strenuous exercise for 24 h before the imaging sessions. On the morning of the PET scan, the participants were instructed to take their usual breakfast, followed by a 4-h fast. Upon arrival at the imaging center, a venous catheter was placed in each forearm for tracer injection and blood sampling, respectively.

^11^C-AcAc PET scans were obtained under four conditions (Figure 1): a 4-h fast (“fasted”), a 4-h fast + D-BHB, fed, and fed + D-BHB. The fed condition consisted of drinking a bottle of a complete liquid meal (Boost^®^ Original; Supplementary Table S1), which was consumed 20 min before the scan. The oral D-BHB consisted of a single 12 g dose of D-BHB dissolved in 150 mL water (Supplementary Table S1) and was given 30 min before the PET scan. The experimental conditions and imaging sessions were scheduled at least 1 week apart. The first three conditions were randomized, and the fourth was added following preliminary data analysis. Blood samples for clinical chemistry were taken during the PET scans.

### Imaging protocol

All PET scans were performed on a Biograph Vision 600 scanner (Siemens, Erlangen, Germany) with a 26-cm axial field of view. A 30-min, cardiac-gated, list-mode PET acquisition centered on the heart and kidney was performed, starting with the intravenous injection of ^11^C-AcAc (341 ± 70 MBq). Blood samples were taken 3, 6, 12, 22, and 28 min post-injection to scale the image-derived input function and measure the ^11^C-CO_2_ concentration for metabolite correction (Ng et al., 2013). A two-pass whole-body acquisition was then performed to visualize radiotracer distribution.

Dynamic and cardiac-gated images were reconstructed from the list-mode acquisition using an iterative algorithm with point spread function and time-of-flight modeling. For the dynamic reconstruction, time frames were as follows: 12 × 10 s, 6 × 30 s, 6 × 150 s, and 2 × 300 s. The image matrix was 220 × 220 voxels with a voxel size of 1.65 × 1.65 × 3.0 mm^3^. For the cardiac-gated reconstruction, the sum image acquired 5–30 min post-tracer injection was reconstructed into 16 gates with the same matrix and voxel size. The whole-body images had a matrix size of 440 × 440 voxels with a voxel size of 1.65 × 1.65 × 3.0 mm^3^ using the same reconstruction algorithm.

### Image analysis

#### Heart

Left and right ventricular function were analyzed using Cedars-Sinai Medical Center Cardiac Suite software (QPET; Cedars-Sinai, CA, United States). Gated images included only the last 25 min of the scan to avoid the high blood pool signal during the vascular phase of radiotracer distribution. Both ventricles were segmented semi-automatically to derive the end-diastolic volume, end-systolic volume, ejection fraction, and myocardial mass.

Cardiac kinetic modeling was done using the PMOD 3.9 cardiac module (PMOD Technologies LLC., Zurich, Switzerland). The ventricles were segmented semi-automatically, and the arterial input function was derived from the left-ventricle cavity. The resulting time–activity curves (TACs) were compared using the following semi-quantitative parameters: peak standardized uptake value (SUV_peak_), time-to-peak (T_peak_), and area under the curve (AUC).

**FIGURE 2:**
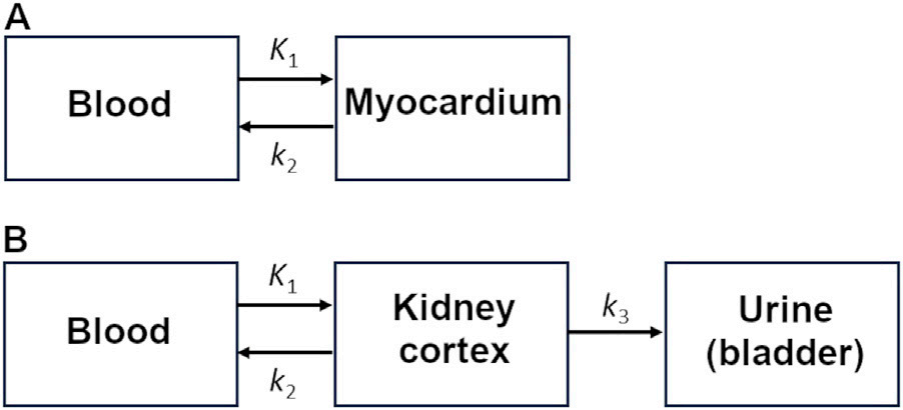
Kinetic models for the **(A)** heart and **(B)** kidney.

For myocardial kinetic modeling, a one-tissue, two-compartment model with built-in partial-volume correction was fitted over the first 15 min of the scan, as previously reported (Figure 2A; Cuenoud et al., 2023). Metabolite correction was based on the measured ^11^C-CO_2_ concentration from blood samples. The metabolic rate constants were *K*_1_ (mL/g/min), representing the radiotracer uptake by the tissue, and *k*_2_ (min^-1^), representing ^11^C-CO_2_ tissue clearance as a surrogate for oxidative metabolism. *K*_1_ was divided by the rate–pressure product (RPP) to normalize the perfusion results to cardiac workload (Murthy et al., 2018).

#### Kidney

The kidney cortex was delineated manually on a summed image of the radiotracer washout period (2–30 min), taking care to avoid contamination from the signal in blood or urine. Kidney TACs were compared using semi-quantitative parameters and kinetic modeling. The three-compartment kidney kinetic model used in the current study (Figure 2B) is similar to that proposed by Hernández-Lozano et al. (2021). It quantifies ^11^C-AcAc uptake (*K*_1_), ^11^C-CO_2_ elimination in the bloodstream (*k*_2_; oxidative metabolism), and urinary excretion of ^11^C-AcAc or other radiolabeled molecules including ^11^C-D-BHB (*k*_3_). It differs from the cardiac model and kidney model previously used by our group (Cuenoud et al., 2023) because it includes possible changes in urinary excretion after a meal or a dose of D-BHB.

The arterial input function and plasma metabolite correction for the kidney were the same as for cardiac kinetic modeling. The total urinary tracer excretion was derived from a region of interest placed over the bladder in the whole-body images. The model was fitted over the first 15 min of the scan.

#### Other organs

Regions of interest for other organs with a visible PET signal, drawn to derive semi-quantitative parameters but without kinetic modeling, included the liver (dynamic and static scan), brain (static scan), and bladder (static scan).

### Laboratory methods

Albumin, aspartate aminotransferase, alanine aminotransferase, creatinine, and high- and low-density lipoprotein cholesterol were measured using commercially available kits on an automated analyzer (cobas; Roche Diagnostics, Indianapolis, United States). The glycated hemoglobin level was measured using the HPLC-723G7 analyzer (Tosoh Bioscience, King of Prussia, PA, United States). The thyroid-stimulating hormone level was measured by sandwich electro-chemiluminescence immunochemistry. Plasma ketones (D-BHB and AcAc), glucose, triglycerides, calcium, magnesium, urea nitrogen, creatinine (Siemens, Deerfield, IL, United States), and free fatty acids (Randox, Kearneysville, WV, United States) in blood sampled during the PET scans were analyzed using automated colorimetric assay (Dimension Xpand Plus; Siemens, Deerfield, IL, United States). C-peptide, gastric inhibitory polypeptide (GIP) (total), glucagon, peptide YY, insulin, and glucagon-like peptide 1 (GLP-1; MilliporeSigma, Oakville, ON, Canada) levels were analyzed using enzyme-linked immunosorbent assay (VICTOR X5, PerkinElmer, Woodbridge, ON, Canada).

### Statistical analysis

Statistical analyses were performed using Prism 9.5.1 (GraphPad Software Inc., CA, United States). All data were expressed as the mean (standard deviation; if data were normally distributed) or median (interquartile range) based on the visual inspection of distribution histograms. Normally distributed data were analyzed using a repeated measures one-way ANOVA with Tukey’s *post hoc* test for multiple comparisons. If not normally distributed, the data were analyzed using the Friedman test with a Dunn’s *post hoc* test for multiple comparisons. Pearson’s correlation coefficients were calculated to assess the relationship between ^11^C-AcAc uptake and factors such as age and blood ketone concentration. A *p*-value ≤0.05 was considered significant.

## Results

### Patient demographics and blood chemistry

The study was conducted on 10 healthy participants with a mean age of 58 ± 9 years, BMI of 25.6 ± 3.3 kg/m^2^, and normal blood chemistry and vital signs (Table 1).

**TABLE 1:** Demographic parameters of the participants.

	n = 10
Male/female	4/6
Age (y)	57.5 ± 9.4
BMI (kg/m^2^)	25.6 ± 3.3
Glucose (mM)	4.7 ± 0.3
eGFR (mL/min)	91.9 ± 9.9
*Vital Signs*	
Heart rate (bpm)	62 ± 6
Diastolic BP (mmHg)	79 ± 12
Systolic BP (mmHg)	129 ± 17

Values are presented as mean ± SD. BP: blood pressure, BMI: body mass index, eGFR: estimated glomerular filtration rate.

### Plasma ketones and ^11^C-CO_2_

**FIGURE 3:**
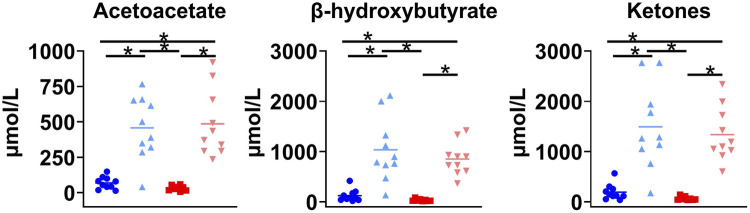
Plasma ketone levels in four conditions. Fasted (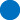), fasted + D-β-hydroxybutyrate (D-BHB) (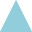), fed (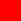), or fed + D-BHB (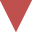). With or without a single dose of D-BHB or a single liquid meal (fed). Fasted: nothing to eat for 4 h. Ketones: AcAc + D-BHB; *p < 0.05.

**FIGURE 4:**
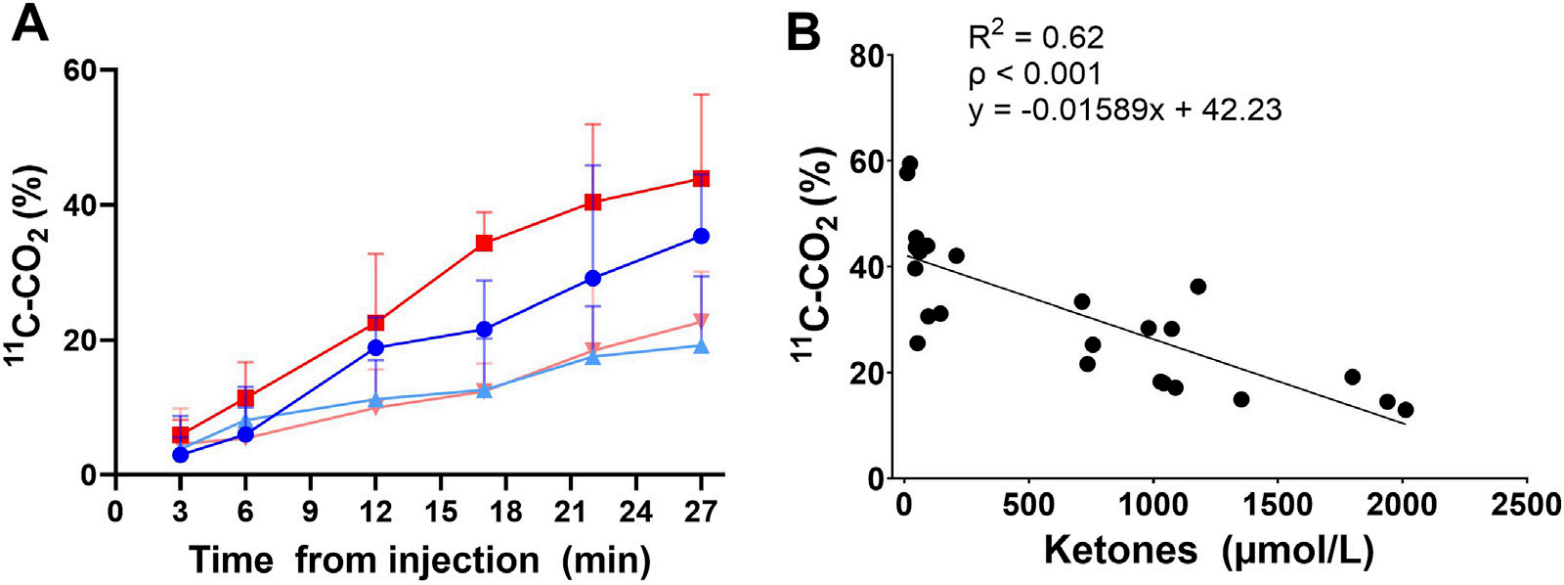
Plasma ^11^C-CO_2_ after ^11^C-AcAc metabolism: rate of ^11^C-CO_2_ increases after the injection of ^11^C-AcAc **(A)**. Fasted (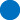), fasted + D-β-hydroxybutyrate (D-BHB) (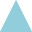), fed (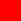), or fed + D-BHB (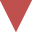). Correlation between the plasma ketone level and plasma ^11^C-CO_2_ 28 min after ^11^C-AcAc injection **(B)**. Data from all conditions were pooled. Ketones = D-BHB + AcAc.

The single oral dose of D-BHB increased plasma total ketones by a mean of 10-fold, increasing both the AcAc and D-BHB levels to a similar extent, irrespective of the fed or fasted condition (Figure 3). The main plasma metabolites of the injected tracer, ^11^C-AcAc, were ^11^C-D-BHB and ^11^C-CO_2_ (Friedemann, 1936). Plasma ^11^C-CO_2_ increased steadily over the 30-min scan period (Figure 4A), so the arterial input function was corrected to remove the contribution of ^11^C-CO_2_ to total radioactivity. There was a greater increase in plasma ^11^C-CO_2_ during the conditions without D-BHB, irrespective of feeding status. The proportion of ^11^C-CO_2_ measured in the blood at *t* = 28 min correlated with total plasma ketone levels (Figure 4B).

The metabolite correction applied to the arterial input function assumed a linear increase in the plasma ^11^C-CO_2_ level and was dependent on individual ketone levels at the beginning of the scan (Equation 1):

$$Cp=Cb-\left(Cb*\frac{\left(\frac{-0.01589*\left[ketones\right]+42.23}{28}*t\right)}{100}\right),$$ (1)

where Cp is the corrected ^11^C-AcAc plasma activity, Cb is the blood ^11^C activity, $\left[ketones\right]$ is the total plasma ketone concentration (µmol/L), and t is the time from injection.

### ^11^C-AcAc PET signal and semi-quantitative parameters

**FIGURE 5:**
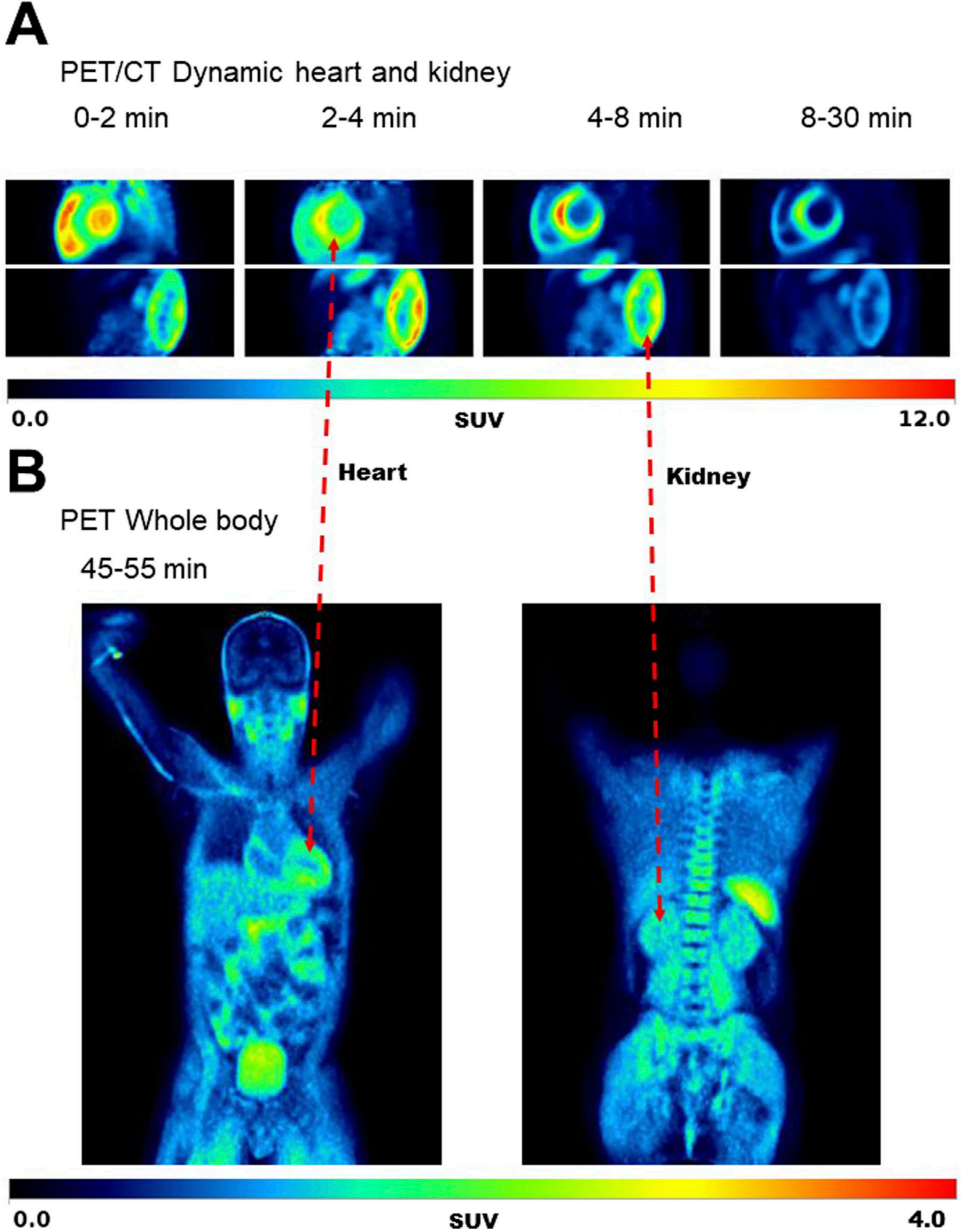
Typical images for the dynamic scan of the heart and kidney **(A)** and whole body **(B)** during the Fasted condition. SUV – standardized uptake value.

**FIGURE 6:**
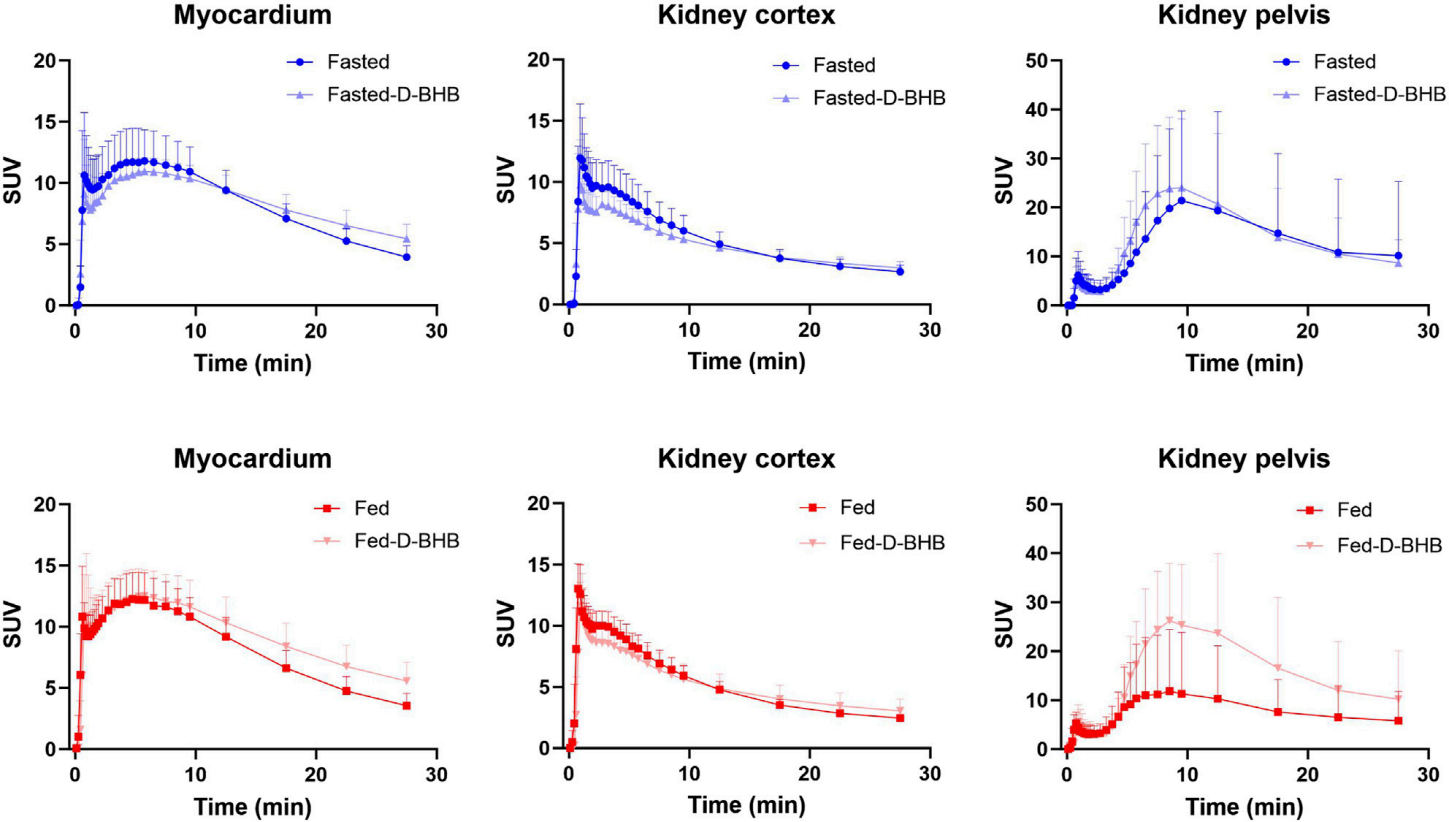
Time–activity curves (mean ± SD) for the myocardium and kidney. D-BHB: single dose of D-β-hydroxybutyrate; fasted: nothing to eat for 4 h; fed: single liquid replacement meal.

Figure 5 shows typical ^11^C-AcAc PET images, and Figure 6 shows the mean TACs for the four conditions. Semi-quantitative features of the TACs are shown in Supplementary Table S2. For the myocardium, significantly slower tracer washout (AUC) was only observed in the fed + D-BHB condition. For the kidney, tracer excretion was significantly higher in the fed + D-BHB condition compared to the fed condition (higher SUV_peak_ and AUC in the pelvis). This difference was mainly due to a lower signal in the fed condition.

In the liver, there was no difference in the signal peak or AUC over the 30-min scan period (Supplementary Figure S1). However, over a longer scan period extended to 45 min, washout of the ^11^C-signal from the liver was slower in the fed and fed + D-BHB conditions. No difference at 45 min was observed for the other organs monitored.

### Heart function

Left ventricular ejection fraction was increased in the fed + D-BHB condition compared to the fasted condition, an effect related to a significantly lower end-systolic volume in the fed + D-BHB condition (*p* = 0.011; Table 2). The end-diastolic volume was higher in the fed condition compared to the fasted + D-BHB condition (*p* = 0.038), an effect compensated for by a nonsignificant increase in the end-systolic volume and no difference in the ejection fraction. No significant difference between conditions was observed in the right ventricular function. The myocardial mass was unchanged by the treatments (Table 2).

**TABLE 2:** Left and right ventricular function.

	Fasted	Fasted D-BHB	Fed	Fed D-BHB
*Left Ventricle*				
Ejection fraction (%)	68 ± 6	71 ± 7	70 ± 6	71 ± 7*
End diastolic volume(mL)	107 ± 24	104 ± 25#	115 ± 25	107 ± 26
End systolic volume (mL)	35 ± 13	32 ± 13	36 ± 13	32 ± 13*
Myocardial Mass (g)	183 ± 34	181 ± 27	189 ± 40	184 ± 36
*Right Ventricle*				
Ejection fraction (%)	67 ± 7	70 ± 9	67 ± 7	69 ± 9
End diastolic volume (mL)	120 ± 21	115 ± 28	125 ± 26	121 ± 26
End systolic volume (mL)	40 ± 14	37 ± 17	42 ± 16	38 ± 17

### Kinetic analysis

**FIGURE 7:**
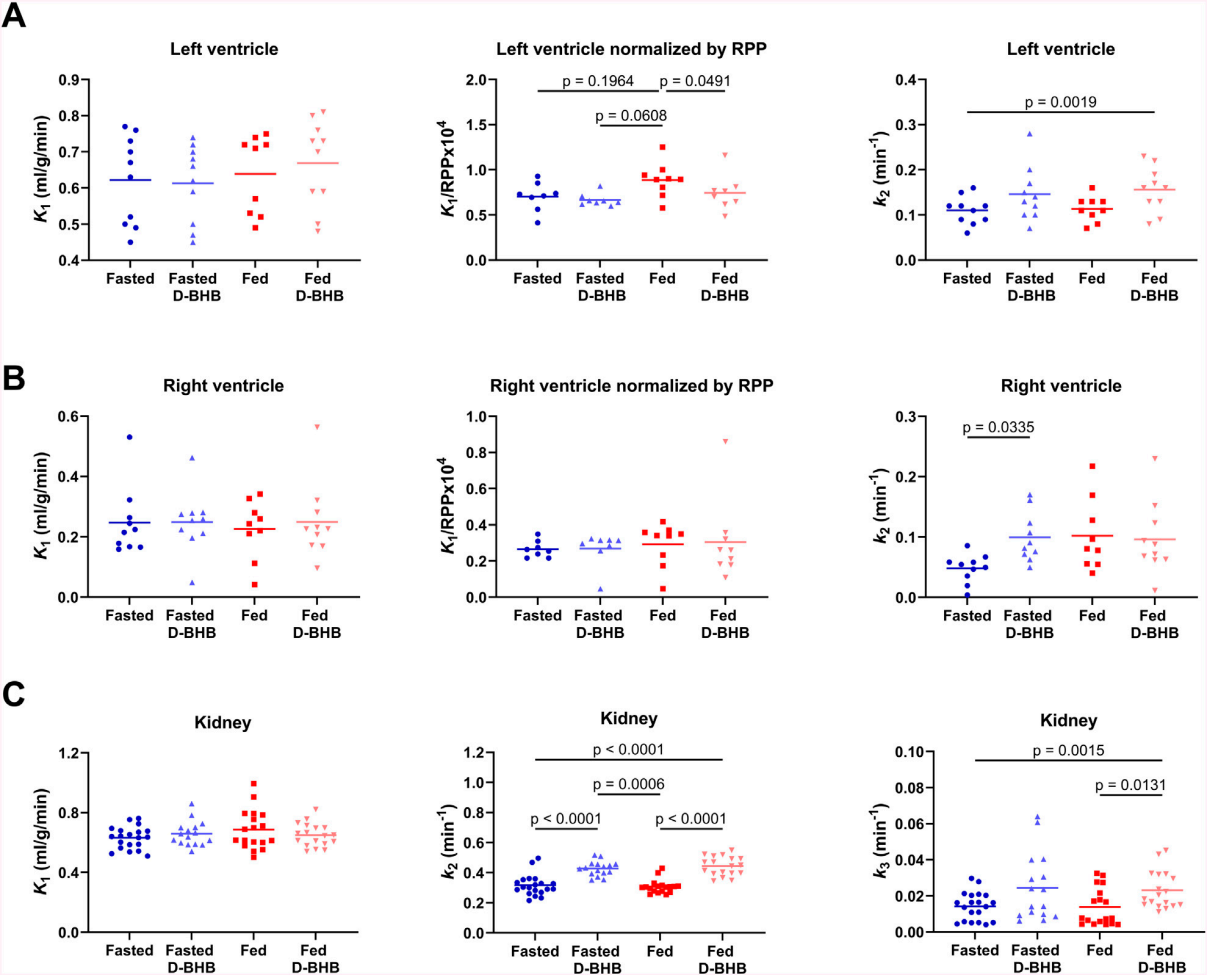
Kinetic parameters for ^11^C-acetoacetate (AcAc). **(A)** Left-ventricle uptake (*K*_1_), uptake normalized by the rate–pressure product (RPP) to account for changes in cardiac workload, and ketone oxidative metabolism (*k*_2_). **(B)** Right-ventricle uptake, uptake normalized by the RPP, and ketone oxidative metabolism. **(C)** Kidney cortex ketone uptake, ketone oxidative metabolism, and urinary excretion (*k*_3_) of ^11^C-labeled compounds. Points for both left and right kidneys are shown. D-BHB: single oral dose of D-β-hydroxybutyrate; fasted: nothing to eat for 4 h; fed: Boost^®^ liquid replacement meal.

Left ventricular kinetic analysis showed no significant difference in ^11^C-AcAc uptake across the four experimental conditions (*K*_1_; Figure 7A). However, after correcting for changes in cardiac workload (*K*_1_/rate–pressure product), *K*_1_ was highest in the fed condition: +17% vs. fed + D-BHB, +23% vs. fasted, and +25% vs. fasted + D-BHB. Moreover, left-ventricle ketone oxidative metabolism (*k*_2_) was also about 35% higher in the fed + D-BHB condition *versus* the fasted condition. A similar trend was observed for fasted *versus* fasted + D-BHB and fed *versus* fed + D-BHB conditions, but the difference was not significant after correction for multiple comparisons. For the right ventricle (Figure 7B), there was no difference in tracer uptake or uptake corrected for cardiac workload. Myocardial tracer oxidative metabolism was 52% lower in the fasted condition compared to the fasted + D-BHB condition (Figure 7B).

There was no difference across the four experimental conditions in ^11^C-AcAc uptake by the kidney (*K*_1_; Figure 7C). D-BHB increased ketone oxidative metabolism (*k*_2_) by 30% in the fasted condition and by 37% in the fed condition. Urinary excretion of ^11^C-labeled compounds was also significantly higher in the fed + D-BHB condition compared to the conditions without the dose of D-BHB.

### Impact of age on heart and kidney ^11^C-AcAc kinetics

**FIGURE 8:**
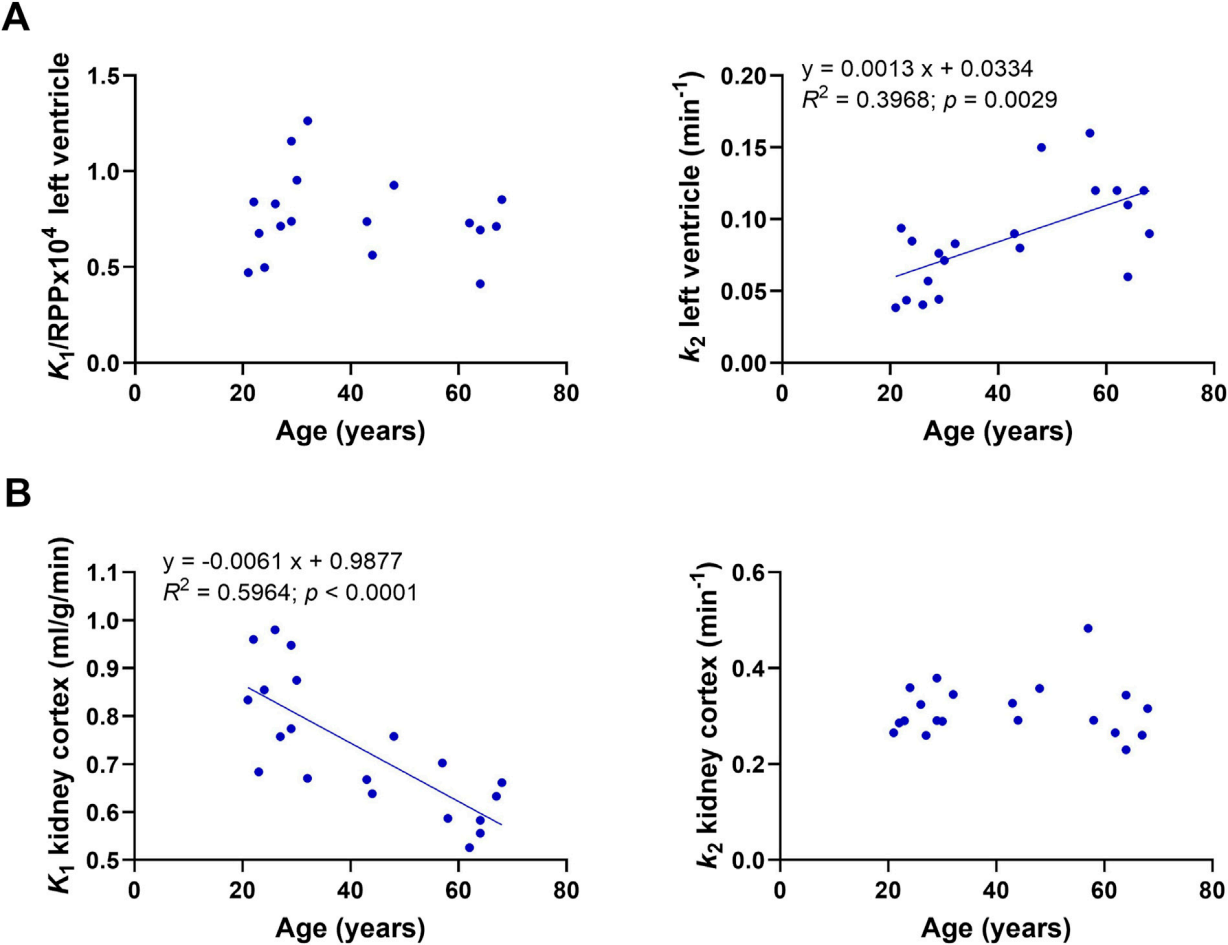
Correlation of kinetic parameters and age. **(A)** *K*_*1*_ and *k*_*2*_ for the left ventricle after a 4-h fast without the single dose of D-BHB. **(B)** *K*_*1*_ and *k*_*2*_ of the kidney cortex after a 4-h fast without the dose of D-BHB.

We previously reported ^11^C-AcAc kinetic parameters in the heart and kidney in healthy adults aged 26 +/- 4 years old (N = 10; Cuenoud et al., 2023). These values were measured after a 4-h fast without the oral dose of D-BHB. By combining those previous data with the present results for older healthy adults in the fasted state, it was possible to assess the effect of age on ^11^C-AcAc kinetics in the heart and kidney (Figure 8). In the myocardium, there was no correlation between age and ^11^C-AcAc uptake (*K*_1_) with or without normalization by the rate–pressure product. In contrast, the kidney uptake of ^11^C-AcAc (*K*_*1*_) was inversely correlated with age. Myocardial oxidative metabolism (*k*_*2*_) of ^11^C-AcAc was significantly positively correlated with age, but no effect of age was observed for *k*_*2*_ in the kidney.

### Hormones and metabolites

Compared to time 0, the fed condition without D-BHB increased plasma glucose, insulin, C-peptide, and GIP levels but decreased blood urea nitrogen levels (Supplementary Figure S2). Compared to the fed condition, fed + D-BHB decreased insulin, GIP, and C-peptide but not glucose levels. Compared to the fasted condition without D-BHB, fed + D-BHB significantly decreased free fatty acids but increased PYY levels. There were no significant differences between treatments for GLP-1 and glucagon (Supplementary Figure S2), triglycerides, magnesium, lactate, or creatinine (data not shown).

## Discussion

Our previous interpretation that ^11^C-AcAc uptake and metabolism by the heart follows a two-compartment model (Cuenoud et al., 2023) is confirmed here, and the present study adds some physiological variables, including the influence of a single meal (fed vs. fasted), an acute dose of D-BHB, and aging. We also report the kinetics of ^11^C-AcAc metabolism by the right ventricle, which, to the best of our knowledge, has not previously been reported.

^11^C-AcAc uptake was similar in the left ventricle as in the kidney, which is different from our previous younger cohort in which the uptake of this tracer was higher in the kidney than in the heart (Cuenoud et al., 2023). However, as in our previous study, the ^11^C-AcAc oxidative metabolic rate was 2–3-fold higher in the kidney than in the myocardium. The three-compartment model was necessary to adequately model kidney ^11^C-AcAc metabolism, including differences in urinary tracer excretion between the study conditions. The three-compartment model reduced the variability in tracer metabolic parameters and had no impact on perfusion values. These data imply the avid metabolism of ketones by both ventricles of the heart and also by the kidney cortex under different nutritional conditions, including a single oral dose of D-BHB. The blood ^11^C-CO_2_ level was inversely correlated with the blood total ketone concentration independent of the fed or fasted conditions, suggesting that whole-body ketone oxidation may be a saturable process.

Tracer uptake (*K*_*1*_) was roughly twice as high in the left ventricle as in the right ventricle under all conditions. Adjusted for cardiac workload (RPP), the left ventricular ketone uptake (*K*_*1*_) was higher in the fed condition, which may be due to postprandial hemodynamic changes driven by insulin (Supplementary Figure S2; Sundell and Knuuti, 2003; Scognamiglio et al., 2005). Interestingly, in the fed condition, left-ventricle ketone uptake increased while insulin, C-peptide, and GIP levels were also increased. With the addition of D-BHB to the fed condition, these parameters resembled the fasted values, implying that ketones may affect the cardiac energy substrate preference, possibly via an effect on insulin secretion.

Several different PET tracers have been used to study kidney function (Toyama et al., 2021; Amoabeng et al., 2022), but these are the first detailed observations on ketone metabolism by the human kidney, as measured by PET imaging with a ^11^C-ketone tracer while taking a single dose of D-BHB. We confirm here the significantly higher ^11^C-AcAc signal in the kidney pelvis than in the cortex (Figure 6; Cuenoud et al., 2023), which is most likely due to the excretion of the tracer and its ^11^C-D-BHB metabolite. Neither the fed nor the fasted condition significantly affected the rate of oxidative metabolism or urinary excretion of ^11^C-AcAc by the kidney, but both measures were increased by the single oral dose of D-BHB (Figure 7C), suggesting that renal ketone oxidation and transport may be increased by higher plasma ketones.

The fed condition is the only one that concomitantly significantly increased insulin, C-peptide, and GIP levels, suggesting that some of the renal transport of ketones might be influenced by changes in these hormones. Interestingly, the renal tubular sodium-coupled monocarboxylate transporter 1 (SMCT1) is a key enzyme in ketone reabsorption (Guo et al., 2023). Insulin reduces SMCT1 activity (López-Barradas et al., 2016), suggesting a possible link between the effect of insulin on renal function and ketone renal reabsorption and excretion (Pina et al., 2020).

The fed condition increased plasma glucose, insulin, GIP, and C-peptide levels, which was in line with previous results obtained with similar mixed meal products (Kössler et al., 2021). In contrast, the oral dose of D-BHB blunted the insulin, GIP, and C-peptide response to the meal but did not change their fasting baseline levels (Supplementary Figure S2). To the best of our knowledge, the inhibitory effect of D-BHB on the GIP response to the meal has not been reported before and merits further study. GIP secretion as a response to nutrient sensing has been implicated in obesity and type 2 diabetes, but its mechanism is complex and involves many signaling pathways (Santos-Hernández et al., 2024).

The left-ventricle uptake of ^11^C-AcAc (*K*_*1*_) was unaffected by age, but its oxidative metabolism (*k*_*2*_) was positively correlated with age (Figure 8A). Both results suggest that ketones are readily metabolized by the myocardium in older healthy adults. The potential impact of increased ketone levels on heart function in the postprandial state may be directly relevant to the lower rate of hospitalization and cardiac sudden death in older people on a sodium–glucose-cotransporter 2 inhibitor, which tends to mildly increase the blood ketone levels (Hlebowicz et al., 2011; Lopaschuk and Dyck, 2023; Xie et al., 2023; Berg-Hansen et al., 2024). Higher plasma ketone levels after D-BHB could, therefore, be beneficial in diseases involving heart failure (Lopaschuk and Dyck, 2023). Unlike in the heart, ^11^C-AcAc oxidative metabolism in the kidney cortex does not appear to change with age. However, kidney ^11^C-AcAc uptake was decreased in older adults (Figure 8B), which could be due to a lower blood flow to the kidney or higher tracer uptake by other organs such as the heart. These observations need to be followed up in conditions in which heart or kidney function may be perturbed or suboptimal.

### Study limitations

This study did not assess the impact of an additional workload on ^11^C-AcAc metabolism by the heart or on ejection fraction. There was relatively low variability in heart and kidney function in these participants, which made it impossible to assess the impact of the different experimental conditions on kidney or heart function. We also neither closely monitored or controlled patient hydration nor used any diuretics, so the urinary output of the participants was highly variable, which could have influenced the renal excretion or reabsorption of ^11^C-AcAc.

PET only measures the presence of the radioisotope itself, so, in the case of ketones, it does not show the extent to which AcAc equilibrates with D-BHB, a parameter which can be observed with hyperpolarized ^13^C-AcAc (von Morze et al., 2018). Differential metabolic processing of ^11^C-AcAc across the kidney pelvis *versus* the cortex was not reported in a different study, in which ^11^C-D-BHB was the ketone tracer, but it may not have been assessed (Luong et al., 2023).

## Conclusion

In this study, cardiorenal ketone metabolism was assessed under acutely different physiological conditions with or without a dose of D-BHB in healthy individuals. The effects of the single meal, the 4-h fast, and the single dose of D-BHB all need validation by longer-term interventions that would be expected to change energy substrate metabolism. This report, therefore, serves as a baseline for further studies involving metabolic manipulation and/or disease conditions affecting the heart or kidney function. For kidney analyses, a three-compartment kinetic model that accounts for variable urinary excretion of the radiotracer was necessary to properly measure changes in ketone metabolism.

## Data Availability

The raw data supporting the conclusion of this article will be made available by the authors, without undue reservation.

## Abbreviations

Ac, acetate; AcAc, acetoacetate; AUC, area under the curve; D-BHB, D-β-hydroxybutyrate; GIP, gastric inhibitory peptide; GLP-1, glucagon-like peptide 1; PET, positron emission tomography; RPP, rate–pressure product; SMCT1, renal tubular sodium-coupled monocarboxylate transporter 1; TAC, time–activity curve.
