# Supplementary material for: Cardiorenal ketone metabolism in healthy humans assessed by 11C-acetoacetate PET: effect of D-β-hydroxybutyrate, a meal, and age
Source: Front Physiol. 2024 Oct 21;15:1443781. doi: 10.3389/fphys.2024.1443781 (PMC11532582; doi:10.3389/fphys.2024.1443781)
Supplement: Supplementary file 1 [file Table1.DOCX]

**The effect of** **D-β-hydroxybutyrate, a meal and age on cardiorenal ketone metabolism:**

**a ^11^C-acetoacetate PET study in healthy humans**

**St-Pierre et al.**

**Supplementary Materials**

**Supplementary Table 1:**

Nutrient information for the Boost® (Original Vanilla) liquid meal (Per bottle; minerals and vitamins not shown) and for the D-beta-hydroxybutyrate (Composition).

| **Per bottle (237 mL)** |  |  |
| --- | --- | --- |
| Kilocalories | 230 |  |
| Carbohydrates (g) | 34 |  |
| Sugars (g) | 14 |  |
| Lipids (g) | 6 |  |
| Cholesterol (mg) | 6.8 |  |
| Monounsaturates (g) | 3.3 |  |
| Saturates (g) | 0.7 |  |
| Protein (g) | 10 |  |
|  | | |

| **Composition** | **Quantity per Sachet** |
| --- | --- |
| ***Medicinal Ingredients*** | |
| Mixture of D-BHB Salts | 15.20 g (as Na-D-BHB [5.75 g], Ca-(D-BHB)2 [6.88 g], Mg-(D-BHB)2 [2.57 g]) |
| D-BHB | 12 g |
|  |  |
|  |  |
|  |  |
| Nicotinamide Riboside Chloride | 0.56 g, providing 0.5 g nicotinamide riboside |
| Excipients 1.98 g | |
|  |  |
|  |  |
|  |  |
|  |  |
|  |  |
| Ca = calcium; D-BHB = Dextro-*beta*-hydroxybutyrate; Mg = magnesium; Na = sodium. | |

**Supplementary Table 2.** Semi-quantitative time-activity curve parameters

|  |  | Fasted | Fasted  D-BHB | Fed | Fed  D-BHB |
| --- | --- | --- | --- | --- | --- |
| Myocardium | **SUV_peak_** | 12.0 (2.6) | 11.2 (1.1) | 12.5 (2.1) | 12.7 (2.3) |
|  | **T_peak_ (min)** | 6.0 (1.8) | 6.0 (1.5) | 5.5 (0.8) | 5.8 (1.1) |
|  | **AUC** | 225 (40) | 230 (20) | 222 (36) | 253 (51)* |
| Kidney cortex | **SUV_peak_** | 10.0 [8.7-11.1] | 8.0 [7.6-9.1]* | 10.7 [9.7-11.2] | 8.5 [7.8-9.6]* |
|  | **T_peak_ (min)** | 2.4 (0.5) | 2.5 (0.5) | 2.5 (0.5) | 2.4 (0.6) |
|  | **AUC** | 142 [124-167] | 135 [126-141] | 145 [133-159] | 126 [120-156] |
| Kidney pelvis | **SUV_peak_** | 14.9 [9.6-38.1] | 25.5 [16.1-40.8] | 9.6 [6.0-18.8] | 29.9 [19.6-42.6]** |
|  | **T_peak_ (min)** | 9.8 (5.8) | 9.2 (3.0) | 7.6 (4.4) | 8.7 (2.5) |
|  | **AUC** | 204 [137-486] | 328 [187-500] | 147 [87-263] | 323 [245-565]** |
| Liver | **SUV_peak_** | 5.6 (1.3) | 5.7 (0.6) | 6.5 (0.8) | 7.0 (2.0) |
|  | **T_peak_ (min)** | 1.9 (0.4) | 1.9 (0.6) | 1.4 (0.2)† | 1.7 (0.3)*** |
|  | **AUC** | 59.5 (11.2) | 64.1 (6.2) | 67.4 (7.8) | 76.4 (21.5) |

Data points are means (±SD) or [interquartile range] * *p* < 0.05; ***p* < 0.01; *p* < 0.001, D-BHB dose vs no D-BHB. † *p* < 0.05, fasted vs fed. SUV_peak_: maximum SUV value, excluding the first-pass bolus; T_peak_: time of the SUV_peak_; AUC: area under the SUV curve.

Liver time-activity curves


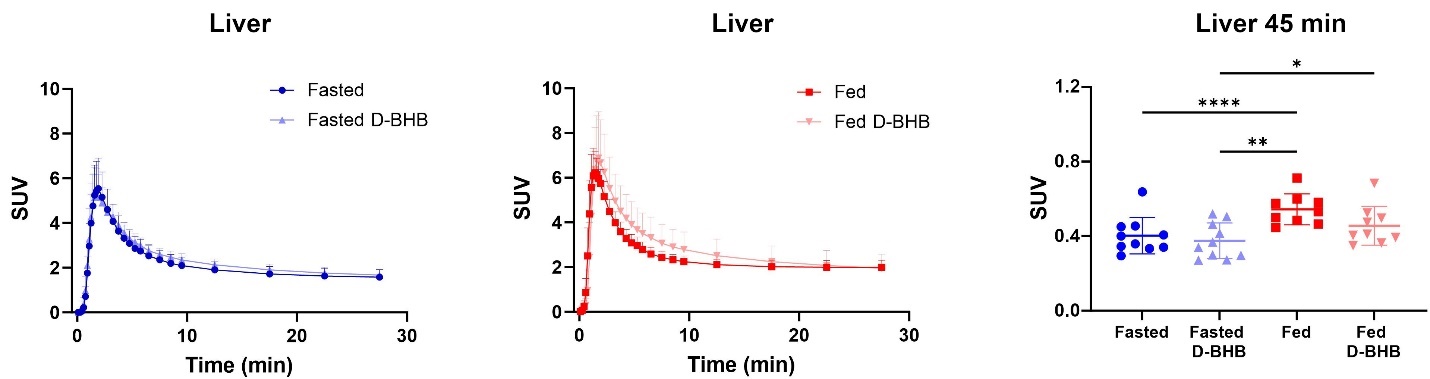


**Supplementary Figure 1.** Time-activity curves for the liver (mean and standard deviation) during the dynamic PET scan (left and center panels), and 45 minutes after injection of the radiotracer - 11C-acetoacetate (static scan; right panel).

# Supplementary Figure 2. Plasma metabolites in four conditions. Fasted (●), Fasted with D-β-hydroxybutyrate (D-BHB) (▲), Fed (■) or fed with D-BHB (▼). Fasted: nothing to eat for 4 h; GIP: gastric inhibitory polypeptide; GLP: glucagon-like peptide 1; PYY: peptide YY; **p* < 0.05.
